# Association between social capital, health-related quality of life, and mental health: a structural-equation modeling approach

**DOI:** 10.3325/cmj.2016.57.58

**Published:** 2016-02

**Authors:** Jafar Hassanzadeh, Mohsen Asadi-Lari, Abdolvahab Baghbanian, Haleh Ghaem, Aziz Kassani, Abbas Rezaianzadeh

**Affiliations:** 1 Department of Epidemiology, Research Center for Health Sciences, School of Health, Shiraz University of Medical Sciences, Shiraz, Iran; 2 Department of Epidemiology, School of Public Health and Oncopathology Research Centre, Iran University of Medical Sciences, Tehran, Iran; 3 Health Systems and Global Populations Faculty of Health Sciences, University of Sydney, Sydney, Australia

## Abstract

**Aim:**

To explore the association(s) between demographic factors, socioeconomic status (SES), social capital, health-related quality of life (HRQoL), and mental health among residents of Tehran, Iran.

**Methods:**

The pooled data (n = 31 519) were extracted from a population-based survey Urban Health Equity Assessment and Response Tool-2 (Urban HEART-2) conducted in Tehran in 2011. Mental health, social capital, and HRQoL were assessed using the 28-item General Health Questionnaire (GHQ-28), social capital questionnaire, and Short-Form Health Survey (SF-12), respectively. The study used a multistage sampling method. Social capital, HRQoL, and SES were considered as latent variables. The association between these latent variables, demographic factors, and mental health was determined by structural-equation modeling (SEM).

**Results:**

The mean age and mental health score were 44.48 ± 15.87 years and 23.33 ± 11.10 (range, 0-84), respectively. The prevalence of mental disorders was 41.76% (95% confidence interval 41.21-42.30). The SEM model showed that age was directly associated with social capital (*P* = 0.016) and mental health (*P* = 0.001). Sex was indirectly related to mental health through social capital (*P* = 0.018). SES, HRQoL, and social capital were associated both directly and indirectly with mental health status.

**Conclusion:**

This study suggests that changes in social capital and SES can lead to positive changes in mental health status and that individual and contextual determinants influence HRQoL and mental health.

Mental health is defined by World Health Organization (WHO) as “a state of well-being in which every individual realizes his/her own potential, can cope with the normal pressures of life, can work productively, and is able to make a contribution to his/her community” (1,2). Mental health and associated disorders have received increasing attention worldwide, largely due to their impact on socio-economic and overall health status of patients (3). Mental health problems remain a global concern, and account for a large fraction of diseases (4,5).

The overall prevalence of mental disorders in Iran between 2000 and 2008 ranged from 12.5% to 38.9% and was similar in urban (20.9%) and rural areas (21.3%) (6). Anxiety and depression were more prevalent than somatization and social dysfunction (7). The provinces with the highest prevalence of mental problems were Chaharmahal with 38.3% and Golestan with 37.3% (8).

Mental health is usually determined by a complex interaction of sociocultural, psychological, environmental, and demographic factors (9). The prevalence of mental health disorders is significantly associated with age, marital status, educational level, employment, and health-related to quality of life (HRQoL) (10). HRQoL incorporates physical and socio-emotional functioning and is used to measure individual's perception of health status, welfare, and well-being in a society (11). A frequently used psychometrical tool for the assessment of HRQoL is Short-Form Health Survey (SF-12). Its two main components are physical component summary (PCS) and mental component summary (MCS), both of which are associated with mental health (12). Previous studies have confirmed a bidirectional association between physical health and depression (as one of the main dimensions of mental health) (13). However, it is not clear whether there is a causal relationship between them (13,14).

The suggested mechanisms by which depression could lead to physical disability and decreased HRQoL are poor health behaviors, increased risk of physical disease, and characteristics of depression (eg, decreased pain threshold) (15). On the other hand, physical disability can lead to depression and deterioration of mental health due to restriction of social activities and loss of social capital (15). Ultimately, this bilateral association between depression and poor physical health can lead to increasing health risks (14).

Mental disorders such as depression and anxiety are also influenced by socioeconomic status (SES) (16). SES is commonly conceptualized as an individual or group’s relative social standing or class (16,17). The main predictors of SES are education level, income, and occupation (15,17,18). The correlations between SES and mental health have been explained by various mechanisms. It has been found that negative impact of low SES on mental health (19) can be reduced by the mediating effect of social capital and physical health (4,18).

Social capital has been defined as individual’s social networks and social interactions, shared norms, values, and understandings that facilitate collective action within or among groups. It can act as a protective factor, promoting mental health status by reducing socioeconomic inequalities (4,20) and play an important role in reducing the prevalence of mental disorders (4). Previous studies have found that social ties and support significantly improve mental health (9). Nonetheless, the association between social capital, mental health, quality of life, and SES is not consistently reported (21,22). This population-based study aims to explore the association between demographic factors, SES, social capital, HRQoL, and mental health among Tehran residents using structural-equation modeling (SEM).

## Methods

### Study design and participants

Data on social capital, HRQoL, and mental health were extracted from a population-based survey entitled Urban Health Equity Assessment and Response Tool-2 (Urban HEART-2), conducted across 22 districts of Tehran in 2011. A multi-stage sampling method was used. All the 22 districts were initially considered as strata (stratified sampling) and we randomly selected 200 blocks from each cluster (district) using a random number generator. Within each block we selected 8 households using systematic random sampling. This resulted in the selection of 31 519 households from 22 districts. Based on an age-sex table, only one person aged 20 years old or more was included from each household (n = 31 519). The study was conducted by more than 500 trained surveyors who were closely monitored for quality assurance.

### Data collection tools

Self-administered package, including social capital questionnaire, mental health questionnaire (General Health Questionnaire, GHQ-28), and health-related quality of life questionnaire (Short Form Health Survey, SF-12) was distributed among male and female participants belonging to four age groups (20-24, 25-45, 46-65, and over 65 years). Previous studies in Iranian population proved the validity and reliability of these instruments: General Health Questionnaire (GHQ-28) (23), the SF-12 questionnaire (12), and social capital questionnaire (24). The GHQ-28, with 28 items, is a common tool for measuring mental health disorders. It has four dimensions – somatic symptoms, anxiety and insomnia, social dysfunction, and depression. The scoring range for each dimension is 0-21, with a score of more than 6 indicating the presence of the disorders. The scoring range for the overall scale is 0-84, with a score of more than 23 indicating some degree of mental health disorders (23).

HRQoL was measured using SF-12v2. This questionnaire is divided into two summary parts including physical and mental components. Its scores range from 0 to 100 (12).

The social capital questionnaire (24) consists of nine subscales, with a total of 69 questions dealing with participants' relationships with family, relatives, friends, neighbors, colleagues, people of the same ethnicity and religion, and general public, with answers on a 5-point Likert scale. The first four subscales measure voluntary participation, collective activity, trust and social cohesion. The fifth subscale involves the items on truthfulness and avoidance of lying, trusteeship, amnesty, fairness and justice, honesty and reliability, and courage to tell the truth. Four other subscales include questions about social support, engagement in association activities (parents-teachers collaboration, religious groups, athletic groups, charities, professional groups, political parties, ethnic groups, and scientific groups), social status (an individual/group’s position in a social structure, based on the cultural, social, economic and other relevant factors), and social networks (an individual’s relationship with family members, relatives, friends, neighbors and colleagues) (24).

Ethical approval was obtained from the Ethics Committee of Shiraz University of Medical Sciences prior to data collection. Participants were explained the aims and scope of the research and all of them gave informed consent. Privacy, confidentiality, and anonymity were respected throughout the study.

### Data analysis

Data were imported into Stata/SE version 12.0 (Stata Corp., College Station, TX, USA) software for detailed analysis. Two main components of models distinguished in SEM are the structural component, showing potential causal dependencies between endogenous and exogenous variables, and the measurement component, showing the relations between latent variables and their indicators (25).

Mental health, HRQoL, social capital, and SES were considered as latent variables and their dimensions viewed as observed variables. All dimensions were used for determining the latent variables in SEM as follows: the four observed variables – somatic symptoms, anxiety and sleep problems, social dysfunction, and depression were used for determining mental health; physical component summary and mental component summary were used for determining HRQoL; individual trust, cohesion/social support, and social trust/associative relationships were used for determining social capital; and the possession (1) or lack (0) of a car, freezer, dishwasher, computer, and a microwave oven, and education status were used for determining SES.

For the purpose of this study, the sequence of considered variables was based on the literature data (4,5,9,10,12). Mental health was considered as the dependent variable and the HRQoL and social capital as the endogenous variables. Other exogenous variables included, SES, age, sex, marital status, job status, family size, living district/neighborhood, and home ownership. The proposed model of associations among these variables was analyzed by Mplus-7 software (Muthén & Muthén, Los Angeles, CA, USA), and variables with no statistical significance (*P* > 0.05) were excluded from the final model. In this model, the correlation between each latent variable and its components (factor loading) and the standardized beta coefficient were calculated.

Descriptive statistics, *t* test, and one-way ANOVA were performed using Stata SE-12 software, and SEM was performed by Mplus-7 software. The goodness of fit of the SEM model was assessed by χ^2^ test, root mean square error of approximation (RMSEA), standardized root mean square residual (SRMR), comparative fit index (CFI), and root mean square residual (RMR). The significance level was set at 0.05. Weighting scheme was also applied for the variables whose distribution was not consistent with the population (the variables that were significant in the χ^2^ goodness-of-fit test). The population based study of Montazeri (12) served as a reference for comparing the variables distribution.

## Results

The mean age was 44.48 ± 15.87 years and family size was 3.52 ± 1.30 individuals. The greatest number of participants were living in Tehran’s district 18 (N = 1619; 5.15%) and the lowest number in district 22 (n = 1027; 3.26%). 35.12% of the participants were male (n = 11 064). The mean mental health score was 23.33 ± 11.10 (range = 0-84). The mean scores for the somatic symptoms, anxiety and sleep problems, social dysfunction, and depression were 6.35 ± 3.79, 6.46 ± 4.81, 8.02 ± 3.69, and 2.50 ± 3.89, respectively. In total, 13 162 individuals (41.76%, 95% confidence interval [CI] 41.21-42.30) had some degree of mental disorder (scores of GHQ-28 > 23). The mean scores for PCS and MCS were 45.35 ± 3.23 and 43.24 ± 4.24 (range = 0-100), respectively. The mean scores for the components of social capital – individual trust, cohesion/social support, and social trust/associative relationships were 3.15 ± 0.65, 2.89 ± 0.66, and 2.15 ± 0.52 (range = 1-5), respectively. Other demographic characteristics of the study participants and the relationships between the demographic characteristics and mental health variables are presented in Table 1.

**Table 1:** Demographic characteristics and mental health status among the residents of Tehran

Variables		N (%)	Mean score of mental health (standard deviation)	*P*
Age	20-30	7369 (23.38)	20.19 (11.21)	0.001*
	30-40	7257 (23.02)	21.30 (11.26)	
	40-50	6023 (19.11)	23.35 (10.79)	
	50-60	5298 (16.81)	23.73 (11.20)	
	>60	5572 (17.68)	26.14 (10.95)	
Sex	male	11,064(35.11)	21.46 (10.73)	0.001**^†^**
	female	20,450(64.89)	24.29 (11.110)	
Nationality	Iranian	31,016(98.68)	21.93 (10.72)	0.001**^†^**
	non Iranian	416 (1.32)	23.36 (11.10)	
Education level	illiterate	2454 (7.83)	25.23 (11.53)	0.018*
	primary school	3034 (9.68)	24.28 (11.29)	
	middle school	4046 (12.91)	23.82 (11.43)	
	high school	13 387 (42.71)	23.55 (10.41)	
	university	8420 (26.87)	21.83 (1.73)	
Marital status	married	23 479 (75.66)	23.28 (10.91)	0.002*
	single	4488 (17.68)	22.71 (11.12)	
	widowed or divorced	2067 (6.66)	25.89 (11.04)	
Employment status	unemployed	1746 (5.59)	25.36(11.96)	0.001*
	student	1605 (5.14)	23.31 (11.04)	
	housewife	15 168 (48.59)	24.13 (11.19)	
	retired	4418 (14.15)	22.42 (10.44)	
	employed	8277 (26.52)	21.13 (10.96)	
House ownership	renting	10 722 (65.91)	24.85 (10.86)	0.013*
	owner	20 733 (34.09)	22.05 (11.51)	

*One-way ANOVA.

†*t* test.

There was almost equal number of participants in each age group, ranging from 16.81% in 50-60 age group to 23.38% in 20-30 age group. Almost half of the participants (n = 13 387, 42.71%) had high school qualifications. The mean mental health score was significantly higher in men than in women (*P* = 0.001). All demographic variables, ie, age, sex, nationality, educational levels, marital status, job status, and house ownership, were significantly associated with mental health (Table 1).

58.24% (n = 18 357) of the participants did not report any symptom of mental disorders. The mean HRQoL (PCS: *P* = 0.001 and MSC: *P* = 0.013) and social capital components (individual trust: *P* = 0.001, cohesion/social support: *P* = 0.018, and social trust/associative relations: *P* = 0.013) were significantly higher in healthy participants than in those with mental disorders (Table 2).

**Table 2:** Associations between health-related quality of life (HRQoL), social capital, and mental health among the residents of Tehran

Variables	Healthy participants (scores <23)		Participants with mental disorders (scores >23)		*P*
	N (%)	Mean (standard deviation)	N (%)	Mean (standard deviation)	
Physical component summary	17 016 (58.36)	47.10 (3.10)	12 139 (41.64)	43.57 (3.36)	0.001
Mental component summary	17 016 (58.36)	44.88 (3.56)	12 139 (41.34)	41.54 (4.22)	0.013
Individual trust	18 280 (58.24)	3.36 (0.62)	13 110 (41.76)	2.96 (0.66)	0.001
Cohesion/social support	18 276 (58.24)	3.05 (0.64)	13 107 (41.76)	2.74 (0.67)	0.018
Social trust/associative relations	18 311 (58.23)	2.24 (0.52)	13 133 (41.77)	2.06 (2.06)	0.013

There was a direct correlation between mental health and dimensions of HRQoL. In addition, the correlation between mental health and social capital components was significant (individual trust: *P* = 0.001, cohesion/social support: *P* = 0.002 and social trust/associative relations: *P* = 0.003). The highest correlation with mental health was found for the component of individual trust (r = -0.48, *P* = 0.001) (Table 3).

**Table 3:** Correlations between health-related quality of life (HRQoL), social capital, and mental health components among the resident of Tehran

Variables	Statistics	Physical component summary	Mental component summary	Individual trust	Cohesion/social support	Social trust/associative relations
Somatic symptoms	Coefficient*	-0.36	-0.42	-0.24	-0.33	-0.10
	*P*	0.001	0.004	0.002	0.031	0.022
Anxiety and sleep problems	Coefficient	-0.29	-0.55	-0.20	-0.14	-0.12
	*P*	0.011	0.010	0.012	0.030	0.012
Social dysfunction	Coefficient	-0.33	-0.50	-0.22	-0.19	-0.13
	*P*	0.001	0.002	0.019	0.003	0.001
Depression	Coefficient	-0.23	-0.26	-0.14	-0.18	-0.26
	*P*	0.002	0.003	0.012	0.009	0.003
Mental health	Coefficient	-0.34	-0.47	-0.48	-0.26	-0.14
	*P*	0.001	0.016	0.001	0.002	0.003

*The negative correlation is due to General Health Questionnaire-28 (GHQ-28) scoring, so that a lower GHQ-28 score indicates a better mental health status.

**Figure 1:**
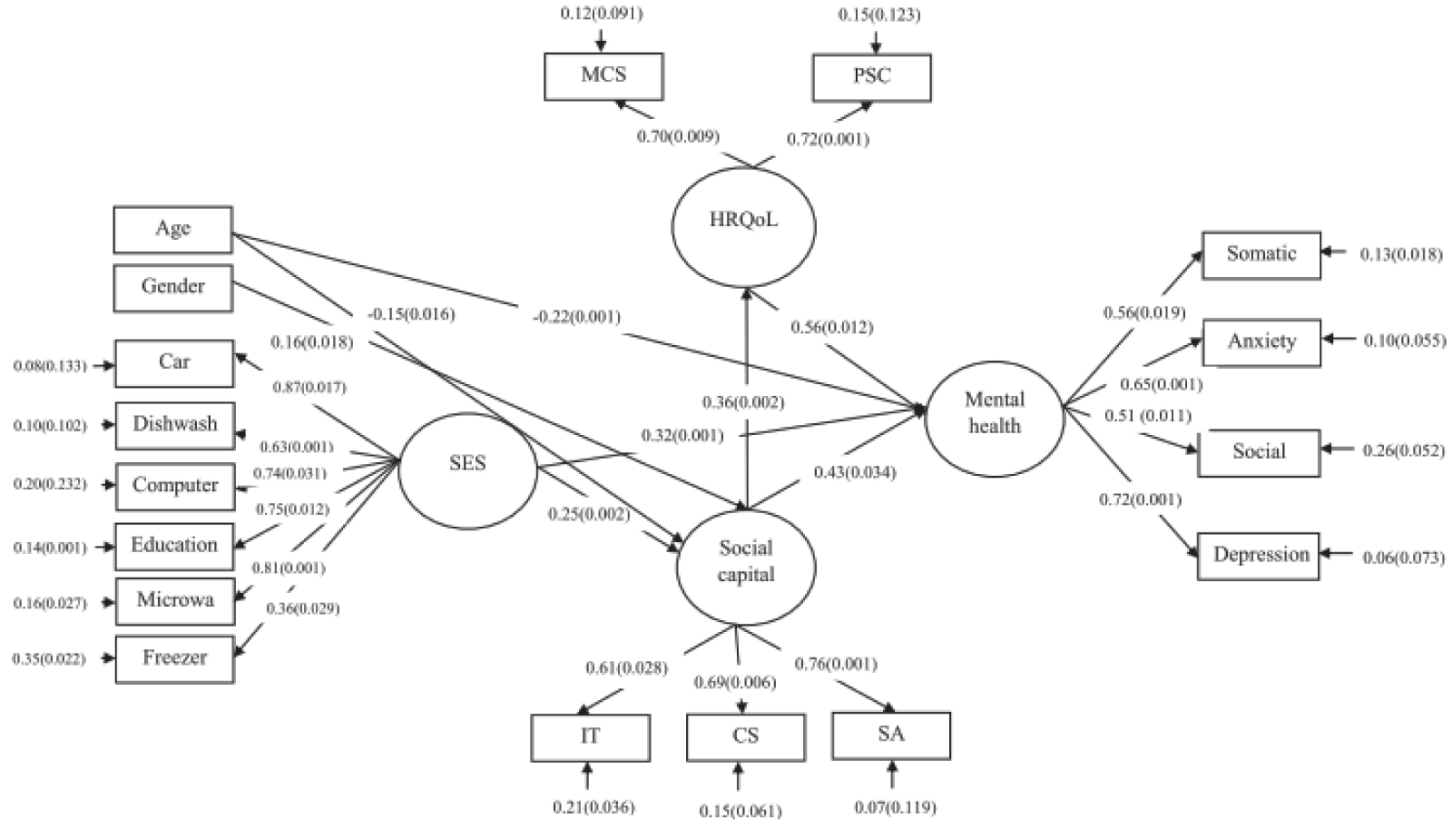
Structural-equation modeling diagram of mental health and associated factors among the residents of Tehran. *IT – individual trust, CS – cohesion /social support, SA – social trust /associative relations, SES – Socio economic Status, HRQoL – Health Related Quality of life, PCS – physical component summary, MCS – mental component summary.

The most powerful determinants of mental health were depression (factor loading = 0.72, *P* = 0.001) and anxiety (factor loading = 0.65, *P* = 0.001). The most powerful determinants of HRQoL were PCS (factor loading = 0.72, *P* = 0.001) and MCS (factor loading = 0.70, *P* = 0.009). The most powerful determinants of social capital were individual trust (factor loading = 0.61, *P* = 0.028), cohesion/social support (factor loading = 0.69, *P* = 0.006), and social trust/associative relations (factor loading = 0.76, *P* = 0.001). The most powerful determinants of SES were ownership of a car, computer, dishwasher, freezer, and a microwave oven, as well as high education levels. Exogenous variables, including nationality, marital status, job status, and home ownership did not show any significant impact on mental health (Figure 1).

Furthermore, direct and indirect association among latent variables was shown by standardized beta coefficient (β). HRQoL, social capital, and SES had a direct association with mental health, while social capital, SES, and mental health through HRQoL had an indirect association. Age and sex were two other exogenous variables that correlated with social capital and mental health. The goodness of fit indices for this model were as follows: χ^2^ = 565, df = 18, *P* = 0.104, RMSEA = 0.02, SRMR = 0.02, CFI = 0.98, and RMR = 0.02.

## Discussion

Our findings indicated that there was an association between social capital and mental health, as well as an association between HRQoL, SES, and mental health. In the univariate analysis, age, sex, educational levels, nationality, job status, and marital status were significantly associated with mental health. Individuals with lower educational levels had a higher prevalence of mental disorders, which is consistent with previous studies (6,26). A possible explanation for this finding could be the poor socio-cultural and economic circumstances among individuals with lower educational levels, and their inability to effectively cope with stressful situations in their life (6,18). We also found a significant correlation between social capital components, PCS, MCS, and mental health. This finding is in line with previous research findings, indicating that mental health promotion within communities can be facilitated by increased social support and cohesion among people (27).

The four dimensions of mental health, somatic symptoms anxiety and sleep problems, social dysfunction, and depression had strong correlations with mental health. The study by Malakouti et al (28) found a correlation between mental health and four factors including depression, psychosocial activity, anxiety, and somatic symptoms.

In our study, social capital variable included components of individual trust, cohesion/social support, and social trust/associative relations. However, other studies investigated other components of social capital, eg, a British Household Panel Survey investigated trust, social participation, civic participation and informal social networks, and social support (29). Discrepancies between the studies may arise from differences in the study design, research methodologies, and instruments applied to measure social capital or mental health.

HRQoL was directly associated with mental health. Social capital was directly and indirectly correlated with mental health through HRQoL. This result is consistent with results from other studies (20,27,30,31). One possible explanation for the association between social capital and mental health is that lower social capital can directly lead to deterioration of mental health by affecting the individuals’ ability to feel satisfied with others, increasing loneliness, and reducing expectations about the future (30,31).

This study also indicated that SES not only directly contributed to individuals’ mental health but that it also affected mental health indirectly through social capital. Other studies also found that that mental health improved with better SES (27,32). SES was also reported to affect physical activity, social capital, and HRQoL, thereby influencing mental health (17). Also, Mielck et al (33) reported that individuals with low SES had greater health impairments and lower HRQoL (33). Two possible explanations for such an association could be a) low SES may lead to mental disorders, and b) mental disorders can restrict an individual’s employment opportunities leading to low SES (16).

Age had a negative, direct association with mental health and it affected mental health indirectly through social capital. Younger individuals reported better social capital and mental health status. A previous study (6) also showed that older age was associated with increased incidence of mental disorders, with individuals over 65 years old having the highest prevalence of mental disorders. The possible reasons of the increased prevalence were retirement, living alone, menopause, and biological factors.

Sex was indirectly associated with mental health through social capital. The negative association between sex and social capital means that social capital level in Iran is higher in men than in women (6). Previous studies in Tehran also found higher prevalence of mental health disorders in women. This may be related to sex roles, environmental factors, work-related stress, social capital, limited social participation, and biological changes (6,31).

A limitation of this study is its cross-sectional design, ie, exploratory variables and mental health were measured simultaneously and we could not obtain any information on how these associations changed over time. Therefore, there is a need for further longitudinal studies, and this population-based study can provide an appropriate basis for such studies. Our study also used pre-designed GHQ-28, which is not a structured clinical interview used for determining the real prevalence of mental disorders. Furthermore, our sample did not include children and adolescents, who comprise a large part of the population. There were more women than men in our sample, probably because at the time of data collection the only family members at home were housewives. We tried to solve this limitation by applying a weighting scheme for the variables that were significant in the χ2 goodness-of-fit test. Despite these limitations, the current study is innovative in its population-based design and its application of SEM approach for determining the associations between the observed and latent variables.

Our findings suggest that changes in social capital and SES can lead to positive effects on mental health status. We also found the influence of individual and contextual determinants on HRQoL and mental health. Enhancing individuals’ education level and providing recreational and social facilities would reinforce SES status and social capital, thus positively affecting mental health.
